# A Novel Method for Treating Bilateral Freiberg's Disease of the Second Metatarsal: A Case Report

**DOI:** 10.7759/cureus.65511

**Published:** 2024-07-27

**Authors:** Aysha Rajeev, George Koshy, Saurav Krishnan, Adithya Panicker, Kailash Devalia

**Affiliations:** 1 Trauma and Orthopaedics, Gateshead Health Foundation National Health Service (NHS) Trust, Gateshead, GBR; 2 General Medicine, Gateshead Health Foundation National Health Service (NHS) Trust, Gateshead, GBR

**Keywords:** chondrogenesis, matrix induced, autologous, bone grafting, bilateral, freiberg`s disease

## Abstract

Freiberg’s infraction is osteonecrosis of the lesser metatarsal heads, most commonly affecting adolescent females. Bilateral Freiberg’s disease is rare, with only a few cases reported. Conservative management is the mainstay of treatment. Surgical management includes the excision of osteophytes and loose chondral flaps, microfracture, corrective osteotomy, and debridement of the metatarsal head, often with unpredictable outcomes.

We report a rare case of a 17-year-old girl with bilateral Freiberg’s disease who was treated with Autologous Matrix-Induced Chondrogenesis (AMIC), achieving excellent radiological and functional outcomes.

## Introduction

Freiberg’s disease is an unusual condition characterized by avascular necrosis of the metatarsal head, primarily affecting the second metatarsal head [1]. It predominantly affects adolescent females and is usually unilateral, with the second metatarsal being the most involved, followed by the third [2]. Bilateral cases are exceedingly rare, with few instances reported in the literature [3,4]. The pathophysiology involves repetitive microtrauma, which leads to disruption of the blood supply to the metatarsal head, which leads to bone death and subsequent collapse of the articular surface. The clinical significance of Freiberg's disease lies in its potential to cause chronic foot pain and altered gait mechanics, which can significantly impact a patient's quality of life and daily activities if left untreated or improperly managed. Non-operative treatment options are limited and include modification of footwear, metatarsal supports, insoles, and intra-articular steroid injections. Operative options include open debridement, cheilectomy, microfracture, and metatarsal neck osteotomy [5,6].

We describe a case of an adolescent female with bilateral Freiberg’s disease treated with a new method, which includes cheilectomy, autologous bone grafting, and the use of an Autologous Matrix-Induced Chondrogenesis (AMIC) membrane, resulting in excellent functional and radiological outcomes.

## Case presentation

A 17-year-old female presented to our foot and ankle unit with a one-year history of pain in her left foot. The pain was insidious in onset and predominantly located around the second toe. She described it as dull, aching, and constant, mainly occurring during weight-bearing activities, which prevented her from participating in any activities for extended periods. There was no history of acute trauma; however, she mentioned a fall a year before the onset of pain. She had tried non-operative treatment modalities without success.

**Figure 1:**
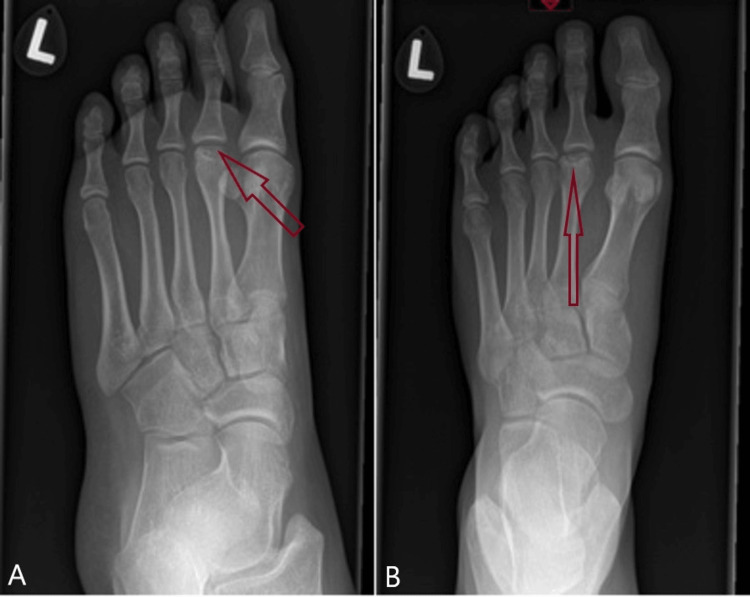
Plain X-rays showing flattening of the head of the second metatarsal are consistent with a diagnosis of Friedberg`s disease A: Oblique view showing flattening of the head of the second metatarsal, consistent with a diagnosis of Friedberg's disease B: Plain AP view showing flattening of the head of the second metatarsal, consistent with a diagnosis of Friedberg's disease

**Figure 2:**
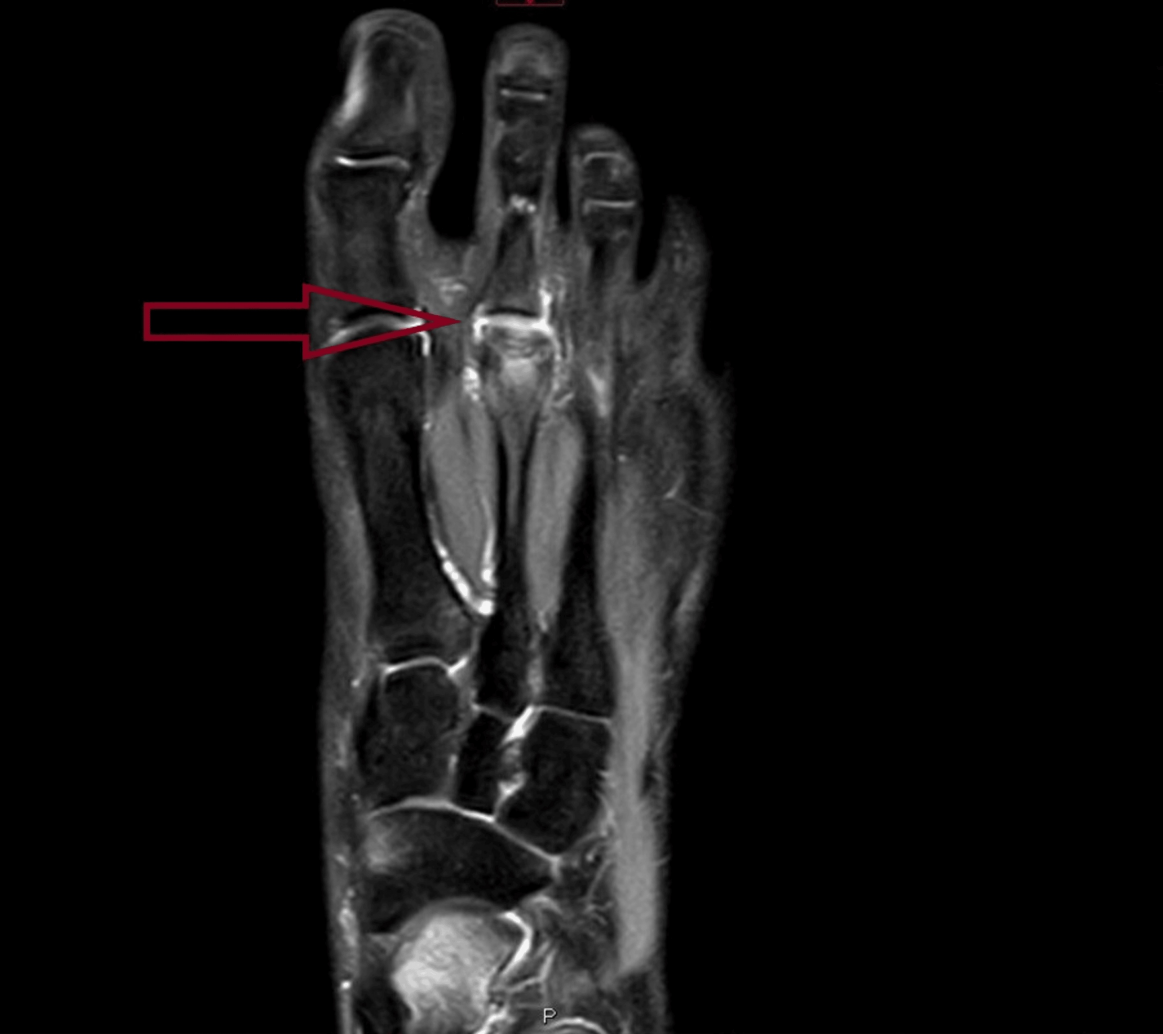
MRI showing collapse of the second metatarsal head with surrounding edema.

On examination, there was swelling on the dorsum of the foot with tenderness around the second metatarsal. Movements of the second MTP joint were restricted due to pain and stiffness. She experienced discomfort during the toe-off cycle of gait. Plain radiography showed flattening of the left second metatarsal head (Figure 1). Magnetic resonance imaging (MRI) revealed the collapse of the second metatarsal head with surrounding edema, suggestive of Freiberg’s disease (Figure 2).

The patient was treated with joint debridement, cheilectomy, calcaneal bone graft, and autologous matrix-induced chondrogenesis (AMIC) using the following surgical technique.

Surgical technique

A dorsal approach to the second metatarsophalangeal joint (MTPJ) was utilized. The head of the second metatarsal was exposed, and unstable articular flaps were removed. Avascular bone tissue was excised to a stable margin. The medullary cavity was opened using a 1.5-mm drill to facilitate angiogenesis. Osteogenesis was induced by autologous bone grafting from the ipsilateral calcaneum. The AMIC membrane (Chondro-Gide) was then applied to the second metatarsal head over the bone graft and fixed with biological glue (Bio-Seal) to induce chondrogenesis.

The post-operative period was uneventful. She was mobilized with heel weight-bearing for the first six weeks, and an active range of motion at the MTP joint was encouraged from day one.

**Figure 3:**
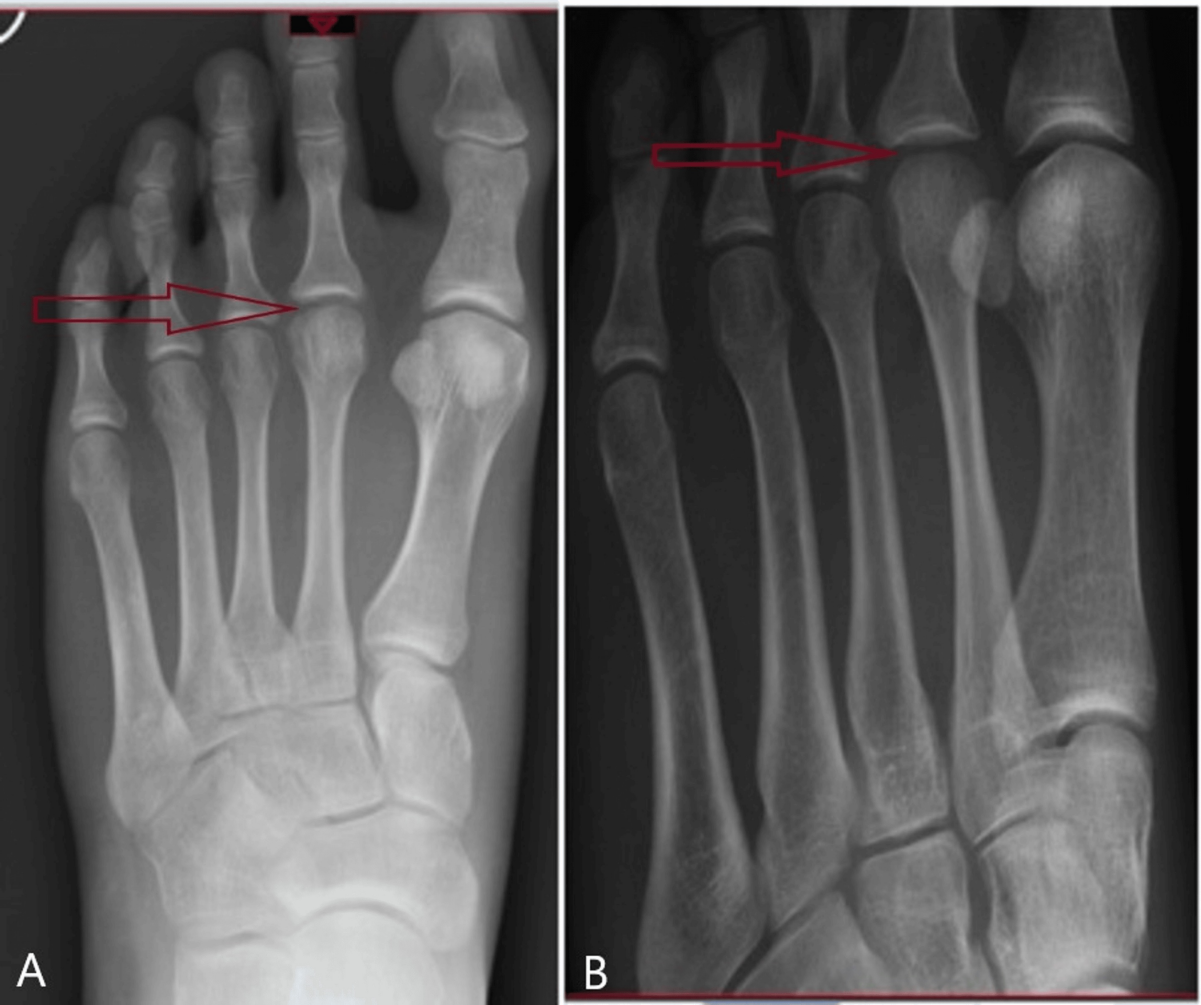
X-rays at the final follow-up showed complete remodeling of the second metatarsal head. A: AP X-ray at the final follow-up showing complete remodeling of the second metatarsal head B: Oblique X-ray at the final follow-up showing complete remodeling of the second metatarsal head

The patient was followed up at intervals of two weeks, six weeks, three months, six months, nine months, and one year. Outcome scores measured using AOFAS (The American Orthopaedic Foot & Ankle Society: Scores range from 0 to 100, with healthy ankles receiving 100 points.) were collected at the three-month follow-up. The patient reported no pain and had returned to all her normal activities. She was discharged at the three-year mark. The X-ray showed complete remodeling of the second metatarsal head (Figure 3). The AOFAS improved from 44 pre-op to 93 at the final follow-up.

**Figure 4:**
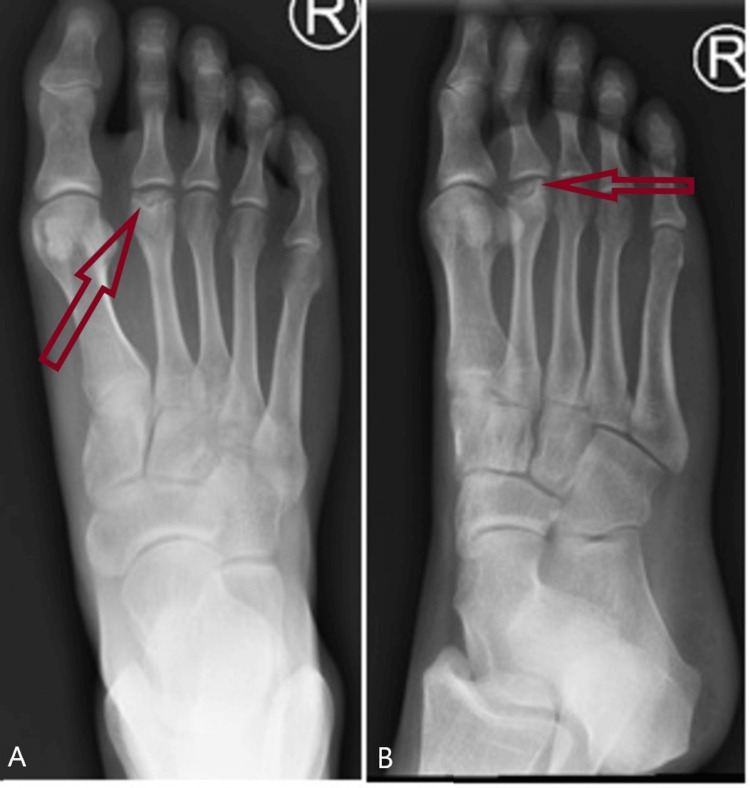
X-rays show flattening of the right second metatarsal head. A: AP X-ray showing flattening of the right second metatarsal head B: Oblique X-ray showing flattening of the right second metatarsal head

**Figure 5:**
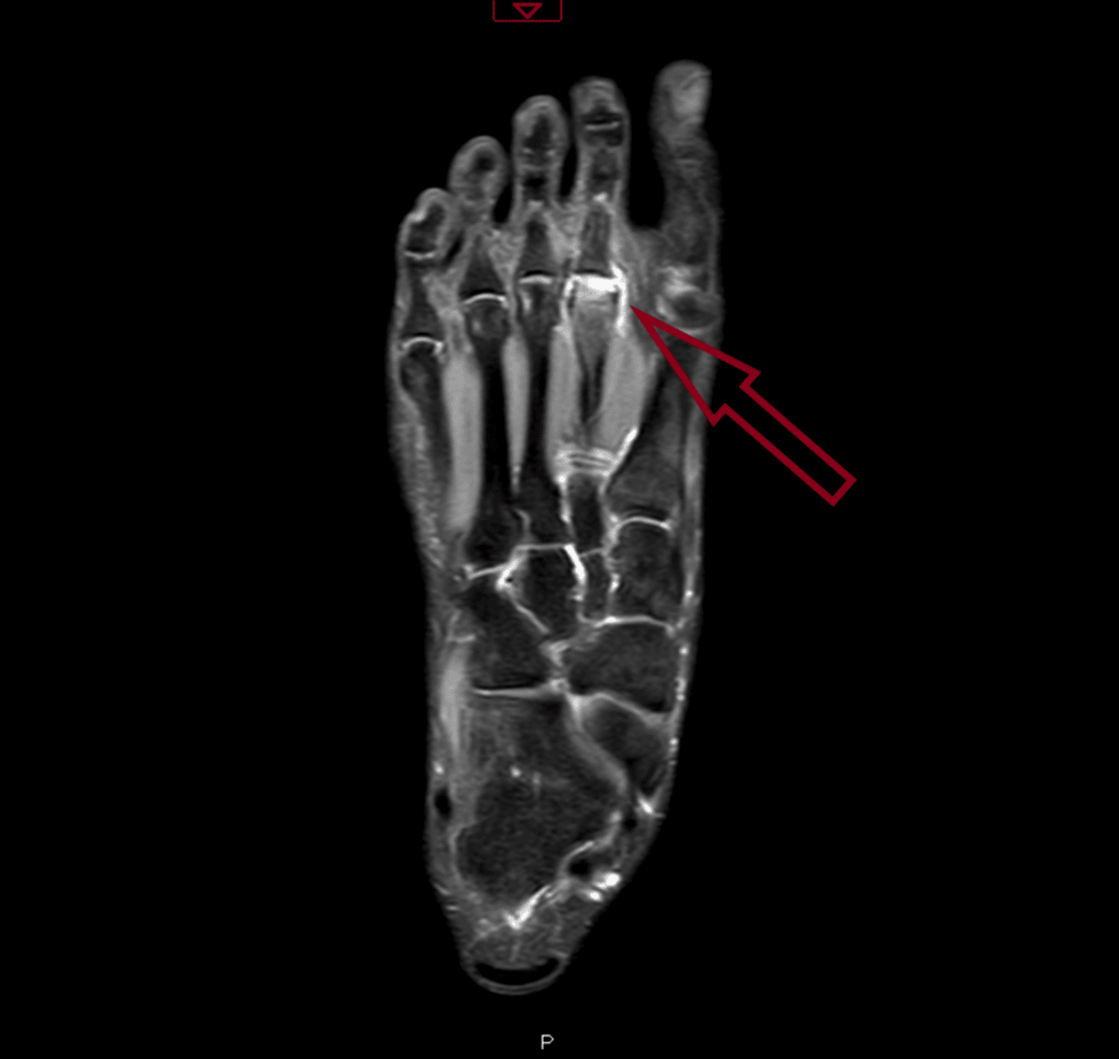
MRI scan showing destruction of the right second metatarsal and surrounding edema.

**Figure 6:**
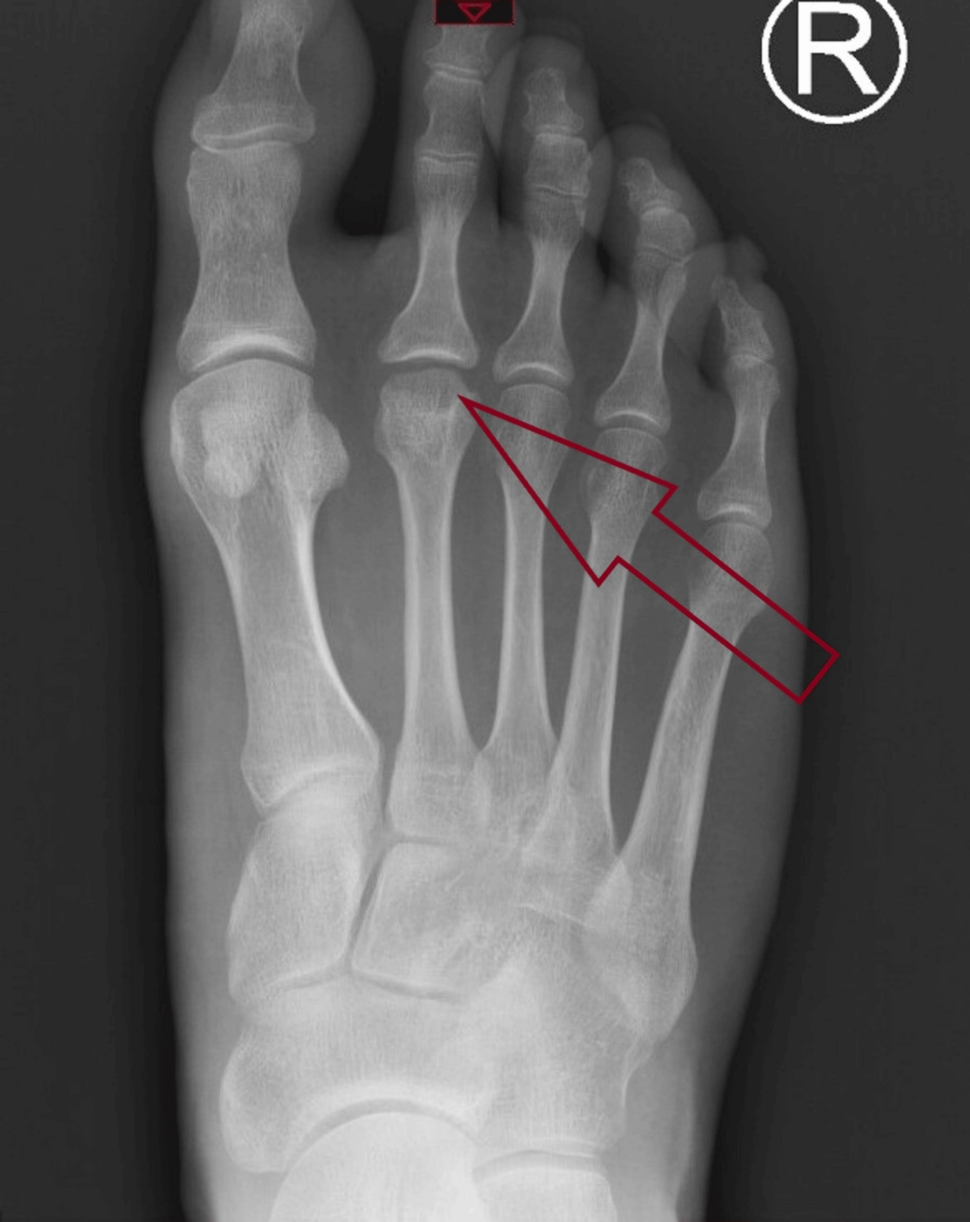
X-rays showed remodeling of the head of the right second metatarsal at the final follow-up.

She presented again one year later with pain in the opposite foot. The nature of the pain was similar to that of the left foot, with no history of trauma. Plain radiography (Figure 4) and MRI (Figure 5) showed features suggestive of Freiberg’s disease. Given her good outcome with the left foot, she was offered similar surgery on the right side. The postoperative period was uneventful. At three months, the patient returned to all her normal activities. At one year, the metatarsal head had started to remodel (Figure 6). The AOFAS improved from 43 pre-op to 95 at the final follow-up at three years.

## Discussion

The etiology of bilateral Freiberg’s disease is unknown. Commonly proposed theories include traumatic and vascular causes, as well as the specific anatomical feature of the second metatarsal being long and relatively immobile, which predisposes it to stress at the metatarsal head [7,8]. Associated systemic conditions include systemic lupus erythematosus, hypercoagulable states, and diabetes mellitus [5,9]. The classification for Freiberg’s disease, proposed by Smillie, is based on findings at the time of open surgery of the affected metatarsals [10] (Table 1). Our patient presented with Stage 3 disease in both feet.

**Table 1:** Smillie`s intraoperative staging system.

Smillie`s stage	Intraoperative findings
1	A fissure fracture in the ischemic metatarsal epiphysis
2	Central portion of the metatarsal head is sunken, altering the contour of the articular surface due to absorption of the underlying bone
3	Furthers sinking of central portion of metatarsal leaving projections on either side due to more bone absorption but plantar articular cartilage remains intact
4	The plantar articular cartilage has given way and the peripheral projections have fractured to form loose bodies
5	Arthrosis with marked flattening and deformity of the metatarsal head

Patients typically present with ongoing pain, difficulty moving the affected metatarsophalangeal joints, and swelling around the involved metatarsal head. The pain is sometimes constant but can be exacerbated by weight-bearing, affecting routine work and social activities [11]. Radiological examinations show widened joint space, flattening of the metatarsal head, fragmentation, and arthritic changes in later stages [12]. In the early stages, MRI scans are useful for diagnosis, revealing flattening of the metatarsal head, sometimes with reversal of convexity, underlying bone edema, loss of cartilage, and subchondral fracture [12].

Conservative management includes footwear modification, insoles, non-steroidal anti-inflammatories, and steroid injections [13,14]. Our patient had tried conservative treatment for more than one year before presenting to our unit. Joint salvage procedures include cheilectomy, metatarsal head dorsal wedge and shortening osteotomies, joint debridement, interposition arthroplasty, and osteochondral autografting or allografting [6,15]. A study by Miyamoto et al. demonstrated good functional outcomes at the end of five years in a series of 13 patients with Freiberg’s disease treated with autologous chondroplasty transplantation (OAT) [16].

Other procedures include hemiarthroplasty and resection arthroplasties. Glazebrook et al. demonstrated good functional outcomes at a 38-month follow-up with a polyvinyl alcohol hydrogel implant for the second metatarsal head [17]. In a case series by Stautberg et al., interposition arthroplasty with a rolled tendon allograft showed improved patient-reported outcomes, high patient satisfaction, and good radiographic outcomes with an average follow-up of 4.2 years [18]. However, Alhadhoud et al. concluded that various surgical options for treating Freiberg’s disease lack sufficient evidence for successful outcomes [6].

Chondro-Gide is a highly refined collagen obtained from pigs, designed to regenerate and mimic human cartilage. It is made of Type I and Type III collagen and is composed of two layers: a smooth, compact top layer and a rough, porous bottom layer. It is compatible with human tissues and can be glued or sutured into the site of chondral defects. The membrane acts as a scaffold to capture mesenchymal cells and growth factors, promoting cell proliferation. The Transforming Growth Factor beta (TGFβ) component of the fibrin glue may contribute to the chondrogenic differentiation of mesenchymal stem cells [19].

Rajeev et al. reported predictable functional and radiological outcomes in a series of 10 patients with Freiberg’s disease treated with microfracture of the osteochondral defect, autologous bone grafting, and the use of an AMIC membrane [20]. We treated our patient by combining these surgical principles to induce neo-angiogenesis, neo-osteogenesis, and eventually neo-chondrogenesis.

## Conclusions

Bilateral Freiberg`s disease is a rare and challenging condition for which the treatment options are very limited. The current operative and non-operative management does not produce good and predictable outcomes. The goal in the treatment of Freiberg`s disease is to achieve pain-free functional movement of the affected metatarsophalangeal joint. Our method of open debridement, micro-fracture, bone grafting, and application of AMIC membrane is an effective surgical option to achieve excellent functional results.
